# The Moderating Role of Close Friends in the Relationship Between Conduct Problems and Adolescent Substance Use

**DOI:** 10.1016/j.jadohealth.2009.12.022

**Published:** 2010-07

**Authors:** Beate Glaser, Katherine H. Shelton, Marianne B.M. van den Bree

**Affiliations:** a The MRC Centre for Causal Analyses in Translational Epidemiology, University of Bristol, Bristol, United Kingdom; b Department of Social Medicine, University of Bristol, Bristol, United Kingdom; c School of Psychology, Cardiff University, Cardiff, United Kingdom; d Department of Psychological Medicine, Cardiff University, Cardiff, United Kingdom

**Keywords:** Adolescent, Substance, Alcohol, Cigarette, Marijuana, Peers, Conduct problems, Prospective, Interaction

## Abstract

**Purpose:**

Conduct problems and peer effects are among the strongest risk factors for adolescent substance use and problem use. However, it is unclear to what extent the effects of conduct problems and peer behavior interact, and whether adolescents' capacity to refuse the offer of substances may moderate such links. This study was conducted to examine relationships between conduct problems, close friends' substance use, and refusal assertiveness with adolescents' alcohol use problems, tobacco, and marijuana use.

**Methods:**

We studied a population-based sample of 1,237 individuals from the Cardiff Study of All Wales and North West of England Twins aged 11–18 years. Adolescent and mother-reported information was obtained. Statistical analyses included cross-sectional and prospective logistic regression models and family-based permutations.

**Results:**

Conduct problems and close friends' substance use were associated with increased adolescents' substance use, whereas refusal assertiveness was associated with lower use of cigarettes, alcohol, and marijuana. Peer substance use moderated the relationship between conduct problems and alcohol use problems, such that conduct problems were only related to increased risk for alcohol use problems in the presence of substance-using friends. This effect was found in both cross-sectional and prospective analyses and confirmed using the permutation approach.

**Conclusions:**

Reduced opportunities for interaction with alcohol-using peers may lower the risk of alcohol use problems in adolescents with conduct problems.

Substance use among young people represents a major public health problem in the United Kingdom [1] and the United States [2]. Cigarette use places a considerable burden on society because of the high rates of associated morbidity and mortality [3,4]. Alcohol meanwhile, is the most prevalent form of substance use during adolescence [1]; its use and abuse have been linked with numerous negative health and social problems, including physical illness, antisocial behavior, poor school performance, violence, and accidents [2,5,6]. Marijuana use remains the most commonly used illicit drug in adolescence [7] and is related to a range of risk factors including lower academic achievement, criminality, and mental health problems [8]. Experimentation with substances is usually initiated in adolescence, yet this is a period of development in which tolerance to substances is lower, and risk of dependence greater, compared to adulthood [9].

Involvement with substance-using peers represents a strong risk factor associated with increased substance use among young people [10–12]. The exact nature of this relationship is not clear; peers may serve as role models, influence personal attitudes toward substance use, and/or provide access, encouragement, and social settings for substance use [13,14]. Best friends, in particular, seem to exert a strong influence on adolescent behavior compared with general friendship networks, or broad-based peer networks [15].

Two major theories have been posited to explain the relationship between adolescents' own substance use and peer substance use: According to the peer cluster model [14], group affiliation is predictive of later adolescent substance use. This places a strong emphasis on group norms and accentuates the active contribution of each peer to the group dynamic [14]. It also posits that a stronger peer context provides more encouragement, access, and rewards for substance use [16]. Alternatively, it is possible that substance use initiation precedes the selection of a substance-using peer-group [17,18] and that substance-using adolescents specifically affiliate with peer groups who match their own behaviors and attitudes (peer-selection model). Finally, both peer selection and peer socialization may occur such that adolescents select their friends according to their own views and behavior, but are also susceptible to peer pressure to conform (bidirectional model) [19].

Some adolescents are likely to be more vulnerable to negative peer effects than others. For example, it has been reported that low refusal assertiveness in interactions with substance-using friends increases the risk that adolescents will themselves become involved with substances [20]. Refusal assertiveness may also mitigate the effect of friends' substance use on adolescents' intention to use substances and their poly-substance use [21].

Irrespective of peer influences, conduct problems in childhood and adolescence have been identified as a major and consistent risk factor for later longer-term use, abuse, and dependence on tobacco, alcohol, and marijuana [12,22,23]. They often precede substance use initiation [23] and may, as a form of early antisocial behavior, represent an important pathway in the development of substance use [24].

The risk of experimentation and progression to heavy use may be particularly increased in adolescents who have multiple risk factors present [6]. However, only few recent studies have evaluated the mechanisms of how multiple risk factors act in concert [20,21] and none of them focused specifically on the interaction between two of the strongest risk factors for substance use—conduct problems and peer influences as exerted by close friends. Although risk for delinquency has been shown to be moderated by peer delinquency [25,26], delinquency measures often do not separate influences on substance use from other deviant behavior. The study of interactions between risk factors, however, can provide invaluable insights into how risk of substance problem use can be exacerbated, attenuated, or even prevented, and is therefore relevant for the development of effective prevention and intervention efforts; especially as there may be gateway effects, with alcohol use preceding the development of illicit drug involvement [27].

A combined effect of behavioral adjustment problems and peer deviance on the risk of substance use and abuse could manifest in different forms. Consistent with the peer cluster model [14] and the theory of reciprocal causation [19], it could be hypothesized that if the relationship between conduct problems and substance use behavior varies as a function of peer affiliation, conduct problems would only be associated with increased risk of substance use in the presence of substance-using friends. By contrast, according to the peer selection model [18,28], no such interaction would be expected, as conduct problems and substance use initiation would precede the affiliation with defiant peers. Thus, conduct problems should represent an independent risk factor for substance use even in the absence of substance-using peers. These relationships may be further moderated by individual-specific traits, such as refusal assertiveness.

The primary aim of this study was to examine the extent to which the concurrent relationship between conduct problems and substance use in adolescence was moderated by close friends' substance use and adolescents' refusal assertiveness. We extended these analyses post hoc to a prospective design and investigated whether similar relationships exist between conduct problems in childhood, and friends' influences and substance use in adolescence.

## Methods

### Sample

This study used data from the Cardiff Study of All Wales and North West of England Twins (CaStANET) which is a population-based twin register including families with twins born between 1976 and 1991 in the Cardiff area of South Wales, and, between 1980 and 1991, twins from the rest of Wales and the North West of England [29]. The sample is representative of the general UK population in terms of socioeconomic status (SES) and ethnicity [29].

For the present study, data was drawn from the second (1996) and fourth wave (2004) of data collection. The sample consisted of 1,237 individuals, who were attending school or college at the time of data collection in 2004 (age range in 1996: 5–10 years; age range in 2004: 11–18 years) and included 530 male (mean = 15.67 years; standard deviation [SD] = 1.88) and 707 female (mean = 15.77; SD = 1.88) respondents from 724 families. The majority of the sample was British/Irish Caucasian (94%). Approximately 73% of the adolescents lived with both their father and mother.

### Measures

*Conduct problems* were assessed in childhood (1996) and adolescence (2004) using mother- and/or self-reports on the adolescent's behavior over the last 6 months. Seven items were adapted from the Strength and Difficulties Questionnaire [30] (see Supplement 1, available online). Items were coded to reflect high levels of behavioral problems. Mother- and self-reported conduct problem scores in 2004 were combined (see Supplement 1, available online; internal consistency α = .80). For participants aged 5–10 years (1996), the questions were answered by mothers only (internal consistency α = .80). All items were added to a total score.

#### Close friends' cigarette, alcohol, and marijuana use

Questions were administered in 2004 and adapted from the Add Health questionnaire [31] (see Supplement 2, available online).

*Refusal assertiveness* was assessed in 2004 using questions adapted from the “Adolescent Substance Use Questionnaire” [32] (see Supplement 3, available online). Answers were recoded to reflect high refusal assertiveness and summed to provide a total score (internal consistency α = .71).

Schools represent a main source for social contacts for adolescents. Young people meet most of their friends through their school, whereas schools can also have strong influences on students' behavioral and social outcomes [33]. It is therefore important to evaluate the relationship between substance use, conduct problems, and peer influences, while adjusting for school influences. *Low school performance* in 2004 within the current school was obtained from combined self- and mother-reports (see Supplement 4, available online). All items were recoded to reflect low school performance and added to a total score (internal consistency α = .82). *Low school satisfaction* in 2004 within the current school (see Supplement 5, available online) was measured using three items, which were recoded to reflect low school satisfaction and combined into a summary measure (internal consistency α = .67).

#### Substance use

Levels of cigarette, alcohol use problems, and marijuana use were assessed in 2004 using self-reported questions derived from the Add Health study [31] (see Supplement 6, available online) and recoded into binary variables as neither of them fulfilled the assumption of normality and only two of them met the assumptions for ordinality.

#### Proxy index of SES

Mothers' report of current financial difficulty in 2004 was used as a proxy measure for SES (see Supplement 7, available online).

### Statistical analysis

#### Data imputation

Imputation was performed by “Imputation-by-Chained-Equations” using STATA (version 11.0) [34]. Imputed values for the missing observations were generated from the posterior predictive distribution using bootstrapping. Five data sets were imputed, for which logistic regression estimates were combined [35].

#### Logistic regression model

Cross-sectional analyses were adjusted for age, sex, SES, school performance, and school satisfaction. The presence of sex-specific increases in log odds of substance use across predictors (interactions) was tested using analysis of covariance and implemented within the logistic regression framework.

Two-way (conduct problems x friends' substance use; conduct problems x refusal assertiveness; peer substance use x refusal assertiveness) and three-way-interaction terms (conduct problems x peer substance use x refusal assertiveness) between predictor variables were specified and investigated with Wald-tests given the non-independence of the data.

Our sample consisted of twin pairs. Because the observations for each twin within a family are not independent, we selected a robust clustered logistic regression model [34] to estimate variances and covariances. This analytical approach allows for family-specific effects, including the influence of sibling behavior, which is another strong predictor of substance use [36]. Before modelling, variables were centered to reduce the potential for collinearity.

However, this approach would not be able to correct for all aspects of genetic and environmental influences that are shared by twin pair members. We therefore used permutations of twin clusters to adjust for shared family- and genetic effects. This approach adjusted also for increased type I error rates often associated with the analysis of multiple interactions [37]. For each permutation, dizygotic and monozygotic twin-pair clusters were permuted separately so that the intraclass-correlation structure of the original sample was maintained. Empirical *p* values were obtained by comparing the statistical evidence for the hypothesized interaction terms in permuted and original data.

## Results

Substance use and predictor variables within our data set contained between .2% and 9.5% missing data across all study subjects. No missing value pattern was observed, and data imputation was performed to increase the power of our analysis.

The overall measures of substance use in our analyses showed that 13.8% of adolescents had smoked, on an average, between one and five cigarettes or more each day in the past month. A total of 46.2% of adolescents reported alcohol use problems over the past 12 months and 16.8% had used marijuana at least once in their lives. Summary measures for all studied variables according to substance use can be found in Table 1. Gender-specific sample characteristics are given in supplement Table S1 (available online). Correlations between substance uses and their predictors can be found in supplement Table S2 (available online) (descriptive analysis only).

### Cigarette use

The odds for cigarette use to noncigarette use were increased by conduct problems and cigarette-smoking close friends, whereas refusal assertiveness skills potentially exerted a buffering effect (see Table 2). No evidence was found for the presence of interactions or for sex-specific effects.

### Alcohol use problems

**Figure 1:**
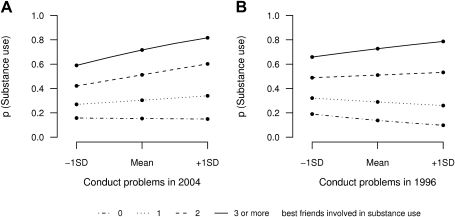
Probability of alcohol use problems. (A) Interaction effect between conduct problems in 2004 and close friends' substance use in 2004. (B) Interaction effect between conduct problems in 1996 and close friends' substance use in 2004. Interaction effects are displayed as probability (*p*) of alcohol use problems across ± 1 SD of the conduct problems measure scale (centered mean), for varying numbers of best friends involved in drinking alcohol (imputed data).

Conduct problems and close friends' alcohol use were identified as risk factors for alcohol use problems, whereas refusal assertiveness had a protective effect. Our results also showed an interaction between conduct problems and close friends' alcohol use (see Table 2), such that the increase in log odds of alcohol use problems per SD in conduct problems was dependent on the number of close friends involved in substance use (see Figure 1A). Higher levels of conduct problems did not affect the probability of alcohol use problems when adolescents had none (OR = 0.97, 95% CI: 0.70–1.35, *p* = .85) or only one close friend using alcohol (OR = 1.18. 95% CI: .94–1.49, *p* = .16). However, a risk effect for conduct problems on alcohol use problems was observed for adolescents with two (OR = 1.44, 95% CI: 1.18–1.77, *p* < .001) or three (OR = 1.76, 95% CI: 1.34–2.30, *p* < .001) close friends using alcohol. However, irrespective of the presence of conduct problems, the presence of at least one alcohol-using friend was significantly associated with alcohol use problems (*p* < .001, data not shown).

Given the possibility that interaction effects may have been detected because of type I error or unaccounted influences of twins on each other, we performed a permutation approach. This confirmed the presence of an interaction effect (empirical *p* value of 0.029; 1,000 permutations) conditional on the information provided by both twins, that is, shared genetic and environmental influences in each family.

We furthermore conducted post hoc analyses to examine relationships across time by analyzing the same constructs but replacing concurrently assessed conduct problems with conduct problems assessed 8 years earlier in childhood to examine the prospective relations between this measure and substance use and peer effects in adolescence (2004). Consistent with our previous findings, the results showed that higher levels of childhood conduct problems did not increase the odds of alcohol use problems in adolescence when adolescents had none (OR = .68, 95% CI: .44–1.05, *p* = .08), one (OR = .86, 95% CI: .65–1.14, *p* = .16), or two (OR = 1.09. 95% CI: .89–1.33, *p* = .40) best friends who used alcohol in adolescence. However, a risk effect was observed for adolescents with three close friends who used alcohol (OR = 1.38, 95% CI: 1.06–1.80, *p* = .02; see Figure 1B) such as indicated by the interaction between childhood conduct problems and close friends' substance use in adolescence (*p* = .01, see Table 3). The nature of these findings was confirmed through sensitivity analysis using one randomly selected twin only (data not shown). Reconstruction of the alcohol problem use measure, omitting questions on “regretting actions because of alcohol” (see Supplement 6, available online) that may reflect underlying conduct problems, revealed similar evidence for a conduct problem x friends' substance use interaction (data not shown). This suggests that the moderating effect is not explained by this item. No evidence was found for interactions involving refusal assertiveness, or for sex-specific effects.

### Marijuana use

Conduct problems and close friends' substance use were identified as risk factors for marijuana use, whereas refusal assertiveness was associated with lower use (see Table 2). There was no evidence for any of the hypothesized interactions nor for sex-specific effects.

## Discussion

This study investigated interaction effects between conduct problems, refusal assertiveness, and close friends' substance use on adolescent substance use. In line with our hypothesis, our findings suggested that the relationship between conduct problems and adolescent alcohol use problems is moderated by their friends' alcohol use.

Substance use is common during adolescence and early adulthood [4]. In the present study, approximately one-seventh of the adolescents had smoked at least one cigarette per day in the past month. Nearly half of all adolescents reported problematic alcohol use during the last year, and approximately one-sixth had taken marijuana at least once in their life. These rates are comparable with published reports of adolescent substance use in the United Kingdom [1,7].

Consistent with previous research, conduct problems and substance use among close friends were strong risk factors for all three substances [6,10–14,22]. In extending these findings, our analysis showed that moderation of their risk effect however is substance-specific. Both cross-sectional and prospective analyses indicated that conduct problems were only related to an increased risk for alcohol use problems in the presence of substance-using friends whereas no such interaction was observed for tobacco and marijuana use. This suggests that the relationship between conduct problems and alcohol use problems is moderated by the peer group, although a greater density of substance-using friends may be needed for the moderation of the link between adolescent alcohol problems and conduct problems in childhood.

Peer influences have recently been highlighted as an important factor in substance use risk moderation, although research to date has mainly focused on interactions with adolescents' personality, including decision-making, self-reinforcement, or refusal assertiveness skills [20,21]. Our results provide evidence for the moderating effect of substance-using close friends that support the peer cluster theory according to which peer affiliation represents an important risk factor for adolescent substance use [14]. We extended this notion by finding that the influences of substance-using close friends may be particularly problematic among adolescents with conduct problems as neither conduct problems in childhood nor in adolescence were risk factors for later alcohol use problems in the absence of substance-using peers.

As conduct problems are related to antisocial behavior [38], our results may reflect a common, transient and adolescent-limited form of antisocial behavior [38]. It has been shown that the adolescence-limited path of antisocial behavior is more strongly associated with delinquent peer affiliation compared to the life-course persistent and pathological path of antisocial behavior [38], and peers may be an important moderating influence on delinquency [26].

Consistent with previous findings [20,21], we also observed a buffering role for high refusal assertiveness on adolescent substance use. The strongest effect was found for cigarette use, followed by marijuana use and alcohol use problems. Previous studies, utilizing a broader definition of friends [20,21], also reported that refusal assertiveness mitigates the influence of friends on adolescents' alcohol use [20], polydrug use [21], and their future smoking intentions [21]. The absence of similar moderating effects within our work may reflect differences in study methodology, in particular the definition of friends as substance-using close friends [15], and the focus on a white European instead of a multiethnic sample.

Alcohol use [1,4], particularly during adolescence, represents common and socially accepted behavior, leaving adolescents particularly susceptible to the influence of alcohol-using close friends. This may also explain why recent research found, in contrast to later tobacco or illicit drug use, no link between early behavior problems and later alcohol use [22], which is consistent with our findings from prospective analysis. However, relationships were identified between childhood conduct problems and early alcohol abuse and dependence [22].

The absence of an effect moderation of conduct problems through friends' influences as observed for tobacco and marijuana use may suggest that these risk factors act independently. This might support the peer selection model [18,28] positing that substance-use initiation precedes the selection of a substance-using peer-group [17,18]. For example, marijuana-using adolescents appear to coordinate their friendships toward congruent values and behaviors [19]. However, given the lower prevalences of tobacco and marijuana use in comparison to alcohol problem use, the absence of interaction effects may also relate to insufficient study power.

The exploration of moderating effects represents an important area of study among theorized risk and protective factors for adolescent substance use, as it has implications for prevention and intervention efforts. The findings of the present study underscore the importance of structured social interventions preventing youth from early exposure to social groups with extensive substance use and/or decreased levels of adult supervision. In addition, our work underlines the importance of training programs to support social competence and refusal skills, as not only peer influences but also individual decisions will determine the outcome of affiliations with deviant peer groups during adolescence [39].

Our study adopted an epidemiological and not a twin study approach as our aims were to assess the moderating effect of the social environment, in particular the interaction with substance-using peers. Although the twin approach enables tests of gene x environment interaction, it is less suitable to address research questions on contextual moderation because of lack of power at the extremes of the moderator [40].

### Limitations

There are several limitations of our study: We utilized adolescents' perceived substance use among close friends as an index of peer substance use and it is possible that the characterization of close friends' behavior is a response artifact because of projection processes [10]. However, despite its potential bias, many studies have shown that perceived substance use is a strong predictor of adolescent peer substance use [6,12]. It would have been beneficial to separate self and peer substance use in time to facilitate tests of direction of effect. However, the consistency of findings assessed concurrently and across time for the interplay between conduct problems and close friends' substance use in the prediction of adolescent substance use suggests that this relationship may be meaningful. Finally, the present findings were based on a sample where some adolescents were still passing through the period of risk for substance use initiation and it will be important to follow this sample into early adulthood and beyond, to examine the developmental course of substance use behavior.

## Conclusions

This study provides further insight into the complex relationship between conduct problems, close friends' substance use, and refusal assertiveness. Cross-sectional and prospective analyses identified a significant moderating effect of close friends on the risk exerted by conduct problems on young people's alcohol problem use. The findings underline the need to consider not only main but also moderating effects, because risk factors appear to act in combination to increase risk of substance use. In addition, we provided further support for the buffering effect of refusal assertiveness and the risk effect of conduct problems and close friends' substance use across all three substances, a finding that has implications for educational and intervention programs.

## Figures and Tables

**Table 1:** Sample characteristics for predictor variables of substance use (imputed data)

Substance use	Predictor	Non-user		User	
		Mean/count	SD	Mean/count	SD
Cigarette use^a^	Gender	469 m;597 f	—	61 m;110 f	—
	Age	15.61	1.87	16.46	1.80
	SES proxy	1^d^	1.02	1^d^	1.09
	Conduct problems in 2004	3.76	2.54	5.51	3.16
	Conduct problems in 1996	1.52	1.80	2.11	2.38
	Low school satisfaction	5.26	1.94	6.44	2.61
	Low school performance	14.19	4.47	17.90	4.93
	Friends' cigarette use	0^d^	0.66	2^d^	1.06
	Refusal assertiveness	14.12	1.32	13.10	2.16

Alcohol use problems^b^	Gender	292 m;374f	—	238 m;333 f	—
	Age	14.92	1.56	16.68	1.77
	SES proxy	1^d^	1.04	1^d^	1.03
	Conduct problems in 2004	3.83	2.64	4.20	2.75
	Conduct problems in 1996	1.60	1.81	1.60	2.01
	Low school satisfaction	5.37	2.00	5.48	2.18
	Low school performance	14.12	4.59	15.38	4.77
	Friends' alcohol use	1^d^	1.12	3^d^	0.92
	Refusal assertiveness	14.11	1.39	13.83	1.61

Marijuana use^c^	Gender	430 m;599 f	—	100 m;108 f	—
	Age	15.53	1.83	16.72	1.79
	SES proxy	1^d^	1.02	1^d^	1.10
	Conduct problems in 2004	3.81	2.57	4.96	3.11
	Conduct problems in 1996	1.55	1.83	1.89	2.20
	Low school satisfaction	5.31	2.01	5.99	2.34
	Low school performance	14.24	4.53	17.00	4.92
	Friends' marijuana use	0^d^	0.37	0^d^	1.04
	Refusal assertiveness	14.10	1.36	13.39	1.98

m = male; f = female; friends = number of substance-using close friends. Estimates for mean and standard deviations (SD) were based on 724 primary sampling clusters (families) and were averaged across multiply imputed data sets.

a 13.8% user.

b 46.2% user.

c 16.8% user.

d Median.

**Table 2:** ORs for substance use (cross-sectional analysis, imputed data)

Substance use	Adolescent behaviour	OR [95% CI]^a^	*p*
Cigarette use^b^	Conduct	1.41 [1.08; 1.85]^c^	.012
	Friends' cigarette use	3.99 [3.18; 5.00]^d^	<.001
	Refusal assertiveness	.62 [.50; .76]^c^	<.001
	Friends x conduct^e^	.96 [.79; 1.17]c,d	.69
	Friends x refusal assertiveness^e^	1.06 [.88; 1.23]c,d	.54
	Conduct x refusal assertiveness^e^	.92 [.76; 1.10]^c^	.35
	Peer x conduct x refusal assertiveness^e^	.87 [.72; 1.04]c,d	.13

Alcohol use problems^f^	Conduct	1.35 [1.10; 1.65]^c^	.004
	Friends' alcohol use	2.41 [2.07; 2.80]^d^	<.001
	Refusal assertiveness	.82 [.71; .96]^c^	.013
	Friends x conduct	1.22 [1.05; 1.41]c,d	.008 (.029)^g^
	Friends x refusal assertiveness^e^	1.01 [.89; 1.13]c,d	.91
	Conduct x refusal assertiveness^e^	.98 [.86; 1.12]^c^	.81
	Peer x conduct x refusal assertiveness^e^	.89 [.79; 1.02]c,d	.095

Marijuana use^h^	Conduct	1.38 [1.10; 1.73]^c^	.005
	Friends' marijuana use	3.67 [2.73; 4.94]^d^	<.001
	Refusal assertiveness	.71 [.60; .84]^c^	<.001
	Friends x conduct^e^	1.21 [.90; 1.62]c,d	.21
	Friends x refusal assertiveness^e^	.81 [.49; 1.32]c,d	.39
	Conduct x refusal assertiveness^e^	.97 [.83; 1.13]^c^	.69
	Peer x conduct x refusal assertiveness^e^	.93 [.80; 1.08]c,d	.35

Conduct = conduct problems (2004); friends = number of substance-using close friends; GOF = goodness of fit.

a Adjusted for sex, age, school performance and school satisfaction and combined across five imputed data sets.

b Fit statistics for cigarette use across data sets: Hosmer-Lemeshow test .99 ≤ p_GOF_ < 1; .41 ≤ Pseudo-R^2^ < .42.

c Per one SD (conduct problems/refusal assertiveness).

d Per one close friend.

e Not included in final model.

f Fit statistics for alcohol use problems across data sets: Hosmer-Lemeshow test .45 ≤ p_GOF_ < .56; .32 ≤ Pseudo-R^2^ < .33.

g Empirical p value based on 1000 permutations.

h Fit statistics for marijuana use across data sets: Hosmer-Lemeshow test .87 ≤ p_GOF_ < .93; .26 ≤ Pseudo-R^2^ < .27.

**Table 3:** ORs for substance use (prospective analysis, imputed data)

Substance use	Adolescent behaviour	OR [95% CI]^a^	*p*
Alcohol use problems^c^	Conduct	1.02 [.83; 1.26]^b^	.84
	Friends' alcohol use	2.56 [2.16; 3.03]^d^	<.001
	Friends x conduct	1.27 [1.06; 1.52]b,d	.011

Conduct = conduct problems (1996); friends = number of substance-using close friends.

a Adjusted for sex and age and combined across five imputed data sets.

b Per one SD.

c Fit statistics across data sets: Hosmer-Lemeshow test .18 ≤ *p*_GOF_ < .25; .31 ≤ Pseudo-R^2^< .33.

d Per one close friend.

## Supplementary Material

Web supplement
