# Supplementary material for: The Moderating Role of Close Friends in the Relationship Between Conduct Problems and Adolescent Substance Use
Source: J Adolesc Health. 2010 Jul;47(1):35–42. doi: 10.1016/j.jadohealth.2009.12.022 (PMC3032885; doi:10.1016/j.jadohealth.2009.12.022)
Supplement: Web supplement [file mmc1.pdf]

Web supplement

S1 Mother- and self-reported conduct problem measures

Adolescents completed the following questions ‘I get very angry and often lose my temper’, ‘I usually do as I am told’, ‘I fight a lot’, ‘I can make other people do what I want’, ‘I am often accused of lying’, ‘I am often accused of cheating’, ‘I take things that are not mine from home, school or elsewhere’. Mothers completed the following questions ‘Often has temper tantrums or hot tempers’, ’Generally obedient, usually does what adult requests’, ‘Frequent fights or is extremely quarrelsome with other young people’, ‘Bullies other young people’, ‘Often tells lies’, ‘Often cheats’, ‘Steals from home, school or elsewhere’(Response options: 0=‘Not true’ to 2=‘Certainly true’). Conduct problems scores in 2004 were constructed by endorsing the highest scores from the adolescent and mother-reports for each item (internal consistency $\alpha = 0.80$) using the “or” rule. Thus, conduct measures in 1996 and 2004 will cover a similar response range. This is a well-established method of endorsement, which has been applied in a range of previous studies e.g. [1,2].

S2 Close friends’ cigarette, alcohol and marijuana use

Adolescents reported on questions about their three best (closest) friends apart from their twin: ‘Of your 3 best friends (apart from your twin), how many smoke at least 1 cigarette a day?’, ‘Of your 3 best friends (apart from your twin), how many drink alcohol at least once a month?’, ‘Of your 3 best friends (apart from your twin), how many use cannabis at least once a month?’ (Response options: 0=‘None’ to 3=‘All three’)

S3 Self-reported refusal assertiveness

Questions included: (1) ‘If my friends were pressing me to do something I didn’t really want to do I would do it anyway’, (2) ‘If my closest friends offered me cigarettes but I didn’t really want them I would smoke them anyway’, (3) ‘If my closest friends offered me drugs but I didn’t really want them I would take them anyway?’ (Response options: 1=‘Strongly disagree’ to 5=‘Strongly agree’).

S4 Mother- and self-reported school performance

Adolescents reported on the questions: ‘I have trouble paying attention in school’, ‘I have trouble getting my homework done’, ‘I do well in exams’ (Response options: 1=‘Not at all’ to 5=‘Very much’), ‘How good is your school or college work?’, ‘According to your teachers, how good is your school or college work?’ (Response options: 1=‘Very poor’ to 5=‘Very good’). Mothers’ report included the questions ‘How good is your child’s school or college work?’ and ‘According to your child’s teachers, how good is his/her school or college work?’ (Response options: 1=‘Very poor’ to 5=‘Very good’). Corresponding mother- and self-reported items were combined as described for conduct problems above.

S5 Self-reported school satisfaction

School satisfaction within the current school was assessed using three self-report items ‘I am happy in my school’, ‘I get along with my teachers’ and ‘I get along with other students’ (Response options: 1=‘Not at all’ to 5=‘Very much’).

S6 Self-reported substance use measures

Cigarette use was assessed with the question, ‘During the past month, on average, how many cigarettes did you smoke each day?’ Response options ranged from 1=‘Never smoked in my life’,2=‘0’, 3=‘1-5’, 4=‘6-10’, 5=’11-20’, 6=’21-30’ and 7=‘More than 30’ and were recoded into ‘Not smoking’ (1,2) and ‘Smoking’(3 to 7).

Alcohol use problems was assessed using three questions A: ‘Over the past 12 months, on how many days did you drink five or more alcoholic drinks in a row?’, B: ‘Over the past 12 months, on how many days have you got drunk on alcohol?’ and C: ‘Have you ever found yourself in situations you later regretted because of alcohol?’ Response options 1=‘Never’, 2=’Once or twice’, 3=’More than twice’, 4=’Often’, 5 =‘Most days’ were combined into ‘No alcohol use problems’(A=1,2, B=1, C=1) and ‘Alcohol use problems’(A,B,C: all other options; internal consistency between all items was α = 0.83). Units of alcohol consumption were defined as a glass of wine, a can or half pint of beer or lager, a bottle (e.g. Bacardi breezer), or a single measure of spirits.

Marijuana use was assessed by the item ‘During your life, how many times have you used cannabis (marijuana/blow/weed/spliff)?’ The six response options ranged from 1=‘Never used cannabis in my life’, 2=‘1-5’, 3=‘6-10’, 4=’11-20’, 5=’21-30’ to 6=‘More than 30’ and were recoded as ‘No marijuana use’(1) and ‘Marijuana use’(2 to 6; once or more lifetime use).

S7 Mother-reported proxy for socio-economic status

This measure was assessed with the question ‘How often are you so short of money you cannot buy the kind of food and clothing that you or your family need?’ (Response options: 1=‘Never’ to 4=‘Frequently’).

References

[1] Costello J, Angold A, Burns, BJ, et al. The Great Smoky Mountains Study of Youth: I. Prevalence and correlates of DSM-III-R disorders. Arch Gen Psychiatry 1996; 53;1137-1143.

[2] Simonoff E, Pickles A, Meyer, JM, et al. The Virginia Twin Study of Adolescent Behavioral Development: influences of age, gender and impairment on rates of disorder. Arch Gen Psychiatry 1997; 54;801-808.

Online supplement tables

S-Table 1: Gender differences in substance uses and their predictors (Imputed data)

		Boys (N = 530)		Girls (N = 707)	
		**Mean/Count**	**SD**	**Mean/Count**	**SD**
**Substance use**	Cigarette use	469nu;61u	-	597nu;110u	-
	Alcohol use problems	292nu;238u	-	374nu;333u	-
	Marijuana use	430nu;100u	-	599nu;108u	-
**Substance use predictor**	Age	15.67	1.87	15.77	1.88
	SES proxy	1#	1.04	1#	1.03
	Conduct problems	4.38	2.87	3.72	2.52
	Conduct problems in 1996	1.90	2.04	1.38	1.76
	Low school satisfaction	5.70	2.17	5.22	1.99
	Low school performance	15.35	4.95	14.21	4.46
	Friends' cigarette use ##	0#	0.88	0#	0.90
	Friends' alcohol use##	2#	1.28	2#	1.24
	Friends' marijuana use ##	0#	0.76	0#	0.46
	Refusal assertiveness	13.93	1.65	14.02	1.39

Note: Estimates for mean and standard deviations (SD) were based on 724 primary sampling clusters (families) and were averaged across multiply imputed data sets; nu – Non-User; u – User; # Median; Friends – Number of substance-using close friends

S-Table 2: Polyserial correlation between substance uses and their predictors (Imputed data)

	Cigarette use	Alcohol use problems	Marijuana use
Gender[#]	0.11	0.034	-0.09
Age	0.24	0.56	0.33
SES proxy	0.16	-0.03	0.08
Conduct problems	0.31	0.09	0.21
Conduct problems in 1996	0.14	-0.001	0.09
Low school satisfaction	0.26	0.03	0.17
Low school performance	0.39	0.17	0.30
Friends' substance use	0.79[1]	0.74[2]	0.70[3]
Refusal assertiveness	-0.28	-0.12	-0.22

Note Estimates of correlations were averaged across multiply imputed data sets; 1 – Tobacco use; 2 – Alcohol use; 3 – Marijuana use; Friends – Number of substance-using close friends; # – Polychoric correlation
